# N-glycosylation of the PEDV spike protein modulates viral replication and pathogenicity

**DOI:** 10.1186/s13567-025-01606-9

**Published:** 2025-08-29

**Authors:** Huixin Zhu, Zhaoyang Feng, Meng Sun, Sinuo Zhang, Zhen Yang, Juan Bai, Ping Jiang, Gongguan Liu, Xing Liu, Xianwei Wang

**Affiliations:** 1 Key Laboratory of Animal Diseases Diagnostic and Immunology, Ministry of Agriculture, MOE Joint International Research Laboratory of Animal Health and Food Safety, College of Veterinary Medicine, Nanjing Agricultural University, Nanjing, 210095 China; 2 Jiangsu Co‑Innovation Center for the Prevention and Control of Important Animal Infectious Diseases and Zoonoses, Yangzhou University, Yangzhou, 225009 China

**Keywords:** Porcine epidemic diarrhea virus (PEDV), spike protein, N-glycosylation, viral replication, pathogenicity

## Abstract

Porcine epidemic diarrhea virus (PEDV), a highly virulent enteric coronavirus, induces severe watery diarrhea and mortality in suckling piglets. The spike (S) protein, a critical mediator of viral entry, undergoes extensive N-linked glycosylation. To elucidate the functional significance of these post-translational modifications, we employed a reverse genetics system to generate 19 recombinant PEDV strains with single-site mutations at predicted N-glycosylation sites. In vitro experiments revealed that mutations at residues N118, N216, N726, N1232, and N1249 significantly attenuated viral replication and reduced plaque size. Our data demonstrated that these mutations impaired viral attachment and internalization. Importantly, in vivo pathogenicity assays in piglets indicated that the N1232Q and N1249Q mutants presented minimal faecal viral shedding and no clinical symptoms, suggesting their potential as live attenuated vaccine candidates. These findings underscore the critical role of S protein glycosylation in PEDV infectivity and virulence, providing a molecular basis for rational vaccine design.

**Supplementary Information:**

The online version contains supplementary material available at 10.1186/s13567-025-01606-9.

## Introduction

Porcine epidemic diarrhea virus (PEDV) is a major pathogen that affects the swine industry and causes significant economic losses globally [1–3]. As a member of the *alphacoronavirus* genus, PEDV infects the intestinal epithelial cells of pigs, inducing gastrointestinal issues such as watery diarrhea, vomiting, and dehydration, which can be fatal in neonatal piglets [4, 5]. To date, PEDV remains a significant threat to the global pig industry [6–8].

The genome of PEDV is approximately 28 kb in length and contains seven open reading frames (ORFs) encoding spike (S), envelope (E), membrane (M), and nucleocapsid (N) proteins and nonstructural proteins [9, 10], among which the S protein is essential for viral entry and infectivity [11–14]. The S protein, a type-I fusion protein, exists as a homotrimer on the virion surface and is composed of two subunits: S1, which is responsible for receptor binding, and S2, which facilitates membrane fusion [15–19]. Given its critical role in viral entry, the S protein is a major determinant of PEDV infectivity and transmissibility [20]. Previous studies have demonstrated that the S gene is one of the virulence determinants of PEDV epidemic strains [21, 22]. Notably, the S protein is extensively glycosylated, and the biological significance of these glycosylation sites warrants further investigation.

N-linked glycosylation is one of the most prevalent post-translational modifications of viral envelope proteins, involving the transfer of oligosaccharides to the asparagine residue within the sequon N-X-T or N-X-S (X refers to all amino acids except proline) in the endoplasmic reticulum (ER) [23, 24]. This modification is crucial for protein folding, stability, and function and plays an essential role in viral entry, intracellular transport and immune evasion [25–27]. For example, the N-glycans on the spike protein of SARS-CoV-2 have been shown to interact with the host cell lectin L-SIGN, which enhances viral entry by promoting receptor clustering and endocytosis [28]. Disruption of the glycosylation of the NS1 protein in flaviviruses leads to abnormal aggregation of the protein, thereby impairing viral replication [29]. In addition, cryo-EM studies have provided high-resolution structural insights into the PEDV spike protein, elucidating the spatial arrangement of its N-glycosylation sites and their potential roles in receptor binding and immune evasion [30–32]. Another study demonstrated that challenging piglets with N-glycan-deficient viruses can substantially diminish the pathogenicity of PEDV [33]; however, the specific glycosylation sites that affect viral virulence remain unknown.

Given the importance of the S protein in PEDV and the role of N-glycosylation in viral biology, we employed a reverse genetics system to generate 19 recombinant PEDV mutants with single-site N-glycosylation deletions in the S protein. Our results demonstrate that specific N-glycosylation sites significantly influence viral replication and pathogenicity, offering valuable insights into PEDV pathogenesis and informing future vaccine design.

## Materials and methods

### Cell lines, viruses, and plasmids

Vero cells (ATCC CRL-1586) and 293T cells (ATCC CRL-3216) were maintained in Dulbecco’s modified Eagle’s medium (DMEM) (Gibco, USA) supplemented with 10% fetal calf serum (Gibco, USA). LLC-PK1 cells (ATCC CL-101) were cultured in MEM (Gibco, USA). All the cells were incubated at 37 °C in a humidified incubator with 5% CO_2_. The PEDV variant strain MSCH (GenBank accession number MT683617, G2b subtype) was isolated and maintained in our laboratory [34]. The WT S protein plasmid was constructed and preserved in our laboratory [35]. N-glycosylation mutant S protein plasmids were generated from the WT plasmid using site-directed mutagenesis and homologous recombination techniques.

### Construction of the full-length infectious clone of the PEDV MSCH

**Figure 1:**
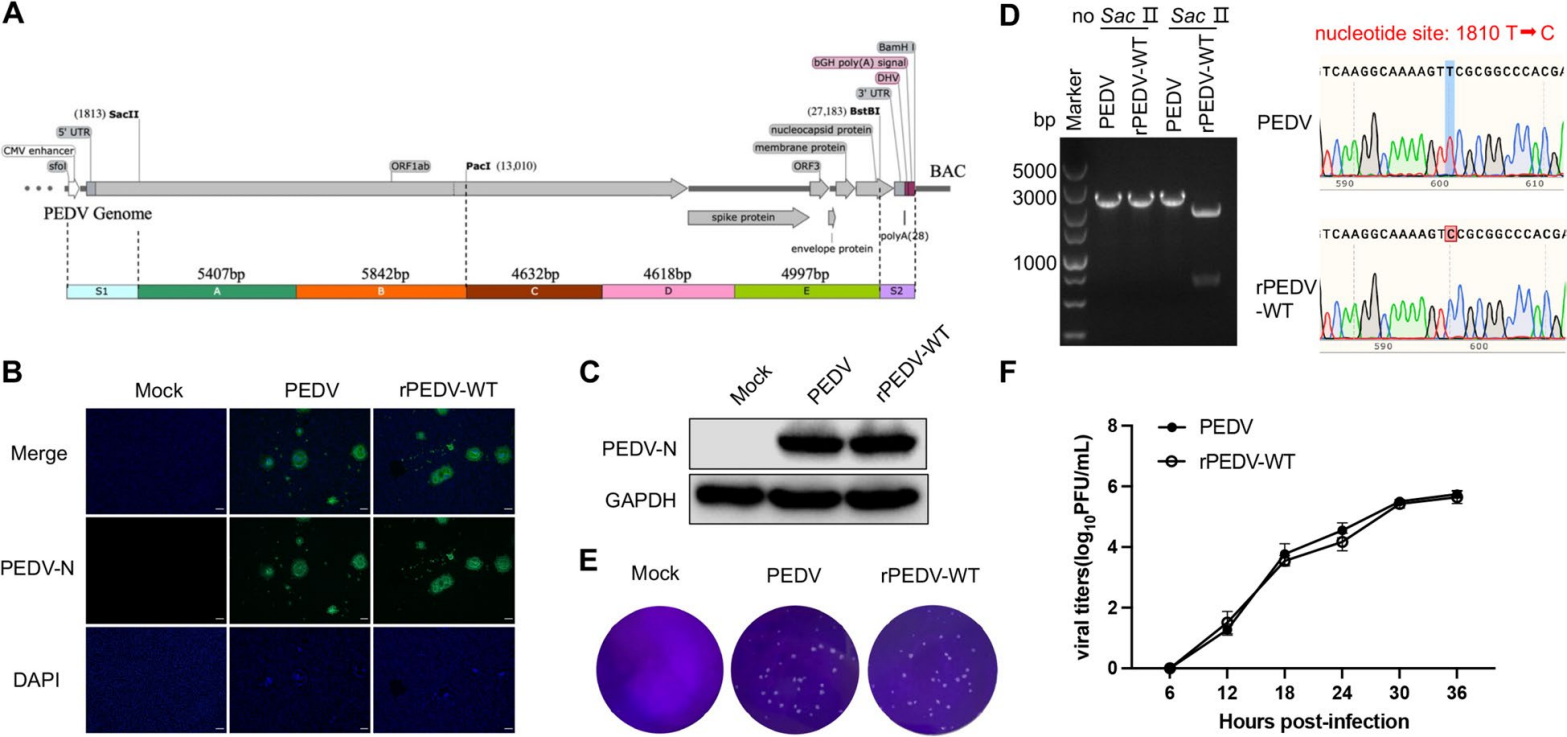
**Construction and rescue of infectious PEDV from the cDNA clone.** **A** Schematic diagram illustrating the construction of a full-length cDNA clone of the PEDV strain MSCH. **B** Vero cells were infected with PEDV or rPEDV (MOI of 0.01). Infected cells were fixed at 16 hpi and immunolabelled with Alexa Fluor 488-conjugated goat anti-mouse IgG. Nuclei were labelled with DAPI (blue). Scale bar, 100 µm. **C** Western blot identified the rescue of rPEDV. **D** The rPEDV RNA of the five-passage virus stock was extracted and reverse transcribed. The rescued virus was confirmed to contain a genetic marker through Sac II restriction enzyme digestion. The marker mutation of rPEDV was subsequently identified by sequencing. **E** Representative images of the plaque morphologies of PEDV and rPEDV. **F** Vero cells in 6-well plates were infected with PEDV and rPEDV. The supernatants were collected at 6, 12, 18, 24, and 36 hpi for titre determination.

A low-copy number BAC vector, pBeloBAC11, was utilized to construct infectious cDNA clones of the PEDV strain MSCH. The BAC vector was modified to incorporate the CMV promoter, PEDV 5′ UTR, the first 800 nucleotides (nts) of ORF1a, the restriction enzyme site of Pac I, nt 26 888–27 701 of the N gene, the PEDV 3′ UTR, a 28-residue poly(A) tail, the hepatitis delta virus (HDV) ribozyme self-cleavage site and bovine growth hormone (BGH) termination sequences (Figure 1A), generating an intermediate BAC plasmid, pBAC-M-PEDV. Total viral RNA was extracted from Vero cells infected with PEDV by using a Viral RNA/DNA Extraction Kit (Omega). cDNA was synthesized from genomic RNA using SuperScript IV Reverse Transcriptase (Invitrogen) following the manufacturer’s instructions. Five overlapping DNA fragments (A: nt 1795–7201; B: nt 7182–13 023; C: nt 12 996–17 627; D: nt 17 608–22 225; E: 22 206–27 202) were amplified with SuperFi™ Green PCR Master Mix (Invitrogen) and then assembled into pBAC-M-PEDV. First, fragments A and B were ligated into pBAC-M-PEDV by homologous recombination using the Infusion Clone Kit (TaKaRa), resulting in the plasmid pBAC-AB. Using the same method, fragments C, D and E were subsequently ligated into pBAC-M-PEDV, yielding the plasmid pBAC-CDE. Finally, the pBAC-AB and pBAC-CDE plasmids were double-digested with *Sac II* and *Pac I* and then ligated together using T4 DNA ligase (Thermo Fisher Scientific) to create the pBAC-PEDV construct.

### Cleavage of pBAC-MSCH and construction of the recombinant BAC

The modification of infectious clones via the CRISPR/Cas9 method was performed as reported by Peng et al. [36]. First, the sgRNA was generated using PrimeSTAR Max DNA Polymerase (Takara) to amplify the sgRNA from the annealed extension of the forward primers sgRNA-1/2/3 and the reverse primer scaffold oligonucleotides into a double-stranded form. The sequences of primers used are listed in Table 1.

**Table 1:** **Sequences of primers used in this study**

Primer name	Sequence (5′ → 3′)	Usage
sgRNA 1F	TTCTAATACGACTCACTATAGGCGCCTGCAGTTGTTGTACGTTTTAGAGCTAG	Construction of sgRNAs
sgRNA 2F	TTCTAATACGACTCACTATAGGCACGCCTAAACCACTTGTGTTTTAGAGCTAG	
sgRNA 3F	TTCTAATACGACTCACTATAGACGCGCAATTATATTATGTGTTTTAGAGCTAG	
Scaffold oligo	AAAAGCACCGACTCGGTGCCACTTTTTCAAGTTGATAACGGACTAGCCTTATTTTAACTTGCTATTTCTAGCTCTAAAAC	
MSCH-1810-F	TACAACAACATGTTCCTGCG	Identification of rescue marker
MSCH-1810-R	GCCAAGCAATTGTAGCTG	
PEDV N-F	TTCTTGTTTCACAGGTGGATG	RT-qPCR detection for PEDV N
PEDV N-R	GCTGCTGCGTGGTTTCA	
mGAPDH-F	CCTTCCGTGTCCCTACTGCCAAC	RT-qPCR detection for GAPDH
mGAPDH-R	GACGCCTGCTTCACCACCTTCT	
pGAPDH-F	TGGTGAAGGTCGGAGTGAAC	
pGAPDH-R	AGTGGAGGTCAATGAAGGGG	

The specific cleavage reaction was performed in a 50 μL mixture containing 5 μg of pBAC-MSCH, 5 μL of Cas9 nuclease (NEB), 20 μg of sgRNAs (10 μg for each sgRNA), and 5 μL of 10 × NEB CutSmart Buffer 3.1 at 37 °C for 4 h. The cleaved pBAC-MSCH was purified using a DNA Cycle Pure Kit (Omega) and verified by electrophoresis in a 0.8% agarose gel. Simultaneously, 21 fragments with N-glycosylation sites AAX (N) mutated to CAG (Q) were prepared through fusion PCR. The linearized BAC fragment was subsequently purified and ligated with the glycosylation mutation fragments by homologous recombination.

### Rescue of infectious PEDVs

Vero cells in six-well culture plates were transfected with BAC plasmids containing the complete or edited genome (4 μg per well) using Lipofectamine 3000 (Invitrogen). At 8 h post-transfection, the cells were rinsed twice with DMEM supplemented with 2 mL of DMEM containing 8 μg/mL trypsin (Sigma). They were then placed in a 37 °C, 5% CO_2_ incubator to facilitate the recovery of the infectious virus. The cells were observed daily for the appearance of cytopathic effects (CPEs).

### Identification of rescue markers

The viral RNA from the fifth-passage (P5) virus stock was extracted and reverse transcribed using the HiScript qRT SuperMix (Vazyme, China). The fragment was amplified by PCR to identify the presence of the markers of rPEDV (nucleotide position 1810) using the primers shown in Table 1. The amplified fragments were digested with the Sac II restriction enzyme and sequenced for identification.

### In vitro replication studies

For the virus attachment assay, wild-type (WT) or N-glycosylation deletion viruses (multiplicity of infection (MOI) of 10) were added to prechilled Vero and LLC-PK1 cells in 24-well plates and incubated for 1 h at 4 °C. The cells were washed twice with cold PBS, and the mRNA levels of PEDV N and GAPDH in the cells were measured using real-time reverse transcriptase quantitative PCR (RT-qPCR).

For the virus internalization assay, Vero and LLC-PK1 cells were infected with WT or N-glycosylation deletion viruses (MOI of 10) at 4 °C for 1 h. The supernatant was replaced with DMEM and then incubated at 37 °C for 30 min. The cells were washed with citrate buffer (pH 3) to remove noninternalized virus. The mRNA levels of PEDV N and GAPDH in the cells were then measured using RT-qPCR.

For the virus replication assay, Vero and LLC-PK1 cells were incubated with PEDV (MOI of 0.01) at 37 °C for 1 h, and the medium was then replaced with fresh DMEM. RT-qPCR was used to measure the mRNA levels of PEDV N and GAPDH in the cell samples at 12 hpi.

### Indirect immunofluorescence assay (IFA)

Vero cells in 24-well plates were infected with PEDV (MOI of 0.01) and incubated at 37 °C for 1 h. After the plates were washed twice with PBS, 500 µL of DMEM containing 8 µg/mL trypsin was added. At 24 h post-infection, the samples were washed three times with PBS, fixed for 15 min with 4% paraformaldehyde, and then permeabilized for 10 min with 0.1% Triton X-100. The fixed cells were incubated with an anti-PEDV N monoclonal antibody (3F10, IgG1 subtype, produced in our laboratory) overnight at 4 °C [37]. After three washes with PBS, the cells were incubated with Alexa-Fluor-488-conjugated goat anti-mouse IgG (Proteintech) for 1 h at 37 °C. The cell nuclei were stained with 4′,6-diamidino-2-phenylindole (DAPI) for 10 min at room temperature. After the samples were washed again with PBS, fluorescence images were obtained using a Nikon A1 confocal microscope (Japan).

### RT-qPCR

Cells were washed once with PBS, and RNA was extracted using the Total RNA Kit I (Omega Bio-Tek, USA) according to the manufacturer's instructions. For reverse transcription, 1 μg of total RNA was reverse transcribed using the HiScript qRT SuperMix (Vazyme, China). The reactions were performed on an ABI QuantStudio 6 System (Applied Biosystems, USA) with AceQ® qPCR SYBR® Green Master Mix (Vazyme, China) according to the manufacturer’s instructions. Relative expression levels of the target genes were calculated via the comparative cycle threshold (CT) method, with host and PEDV levels normalized relative to those of the housekeeping gene. The primers used are listed in Table 1.

### Virus titration

Vero cells were cultured in 6-well plates and allowed to reach confluence overnight. The cells were then incubated with 1 mL of serially diluted supernatant containing PEDV for 2 h at 37 °C and 5% CO_2_. Next, the cells were overlaid with 2 mL of overlay medium (DMEM, 1% low-melting agar, 8 µg/mL trypsin) and incubated at 37 °C and 5% CO_2_ for 72 h. Finally, the cells were stained with crystal violet staining solution (20% ethanol, 2% formaldehyde, 1% crystal violet) for manual plaque counting.

### Cycloheximide (CHX) experiment

293T cells in 24-well plates were transfected with Flag-tagged WT or mutant S plasmids (N118Q, N216Q, N726Q, N1232Q, and N1249Q). Twenty-four hours after transfection, the cells were treated with 100 μg/mL CHX dissolved in dimethyl sulfoxide (DMSO) or with DMSO alone. The cells were collected at 3 and 6 h after treatment and subjected to western blotting.

### Animal experiments

A total of 35 3-day-old piglets from PEDV-free sows were randomly assigned to seven groups: the WT group, N118Q group, N216Q group, N726Q group, N1232Q group, N1249Q group, and control group. Each group contained five piglets. All piglets were confirmed to be free of PEDV, porcine deltacoronavirus, and rotavirus by RT-qPCR analysis of piglet feces before viral challenge. The piglets in each group were challenged with 2 × 10^6^ PFU of the indicated virus or mock-challenged by oral administration. After challenge, daily clinical observations were conducted, and rectal swabs were collected to measure virus shedding using RT-qPCR. At 5 dpc, the piglets were euthanized, and the intestinal segments were collected. These samples were either processed for RT-qPCR analysis to quantify the viral load in the intestines or fixed in 10% neutral-buffered paraformaldehyde for subsequent histopathological examination.

### Pathological examination and IHC of intestine sections

At necropsy, the duodenum, jejunum and ileum tissues were collected and fixed in formalin for histological examination. The dehydrated tissues were cleared with xylene, embedded in paraffin wax, sectioned, and mounted onto slides. These slides were then subjected to hematoxylin and eosin (H&E) staining and immunohistochemistry (IHC) assays. A monoclonal antibody against the PEDV N protein was used as the primary antibody, and an Alexa Fluor 594-labelled goat anti-mouse IgG (Proteintech) was used as the secondary antibody for the IHC procedure.

### Statistical analysis

Data from three individual experiments were analysed using Tukey’s post hoc test for multiple comparisons (GraphPad Prism Software Inc, San Diego, CA, USA) and are presented as mean ± SD (standard deviation). The asterisks in the figures denote significant differences, with *P < 0.05 and **P < 0.01.

## Results

### Rescue of infectious PEDV from the cDNA clone

To investigate the impact of N-glycosylation of the spike protein on PEDV replication and pathogenicity, we employed a reverse genetics approach to generate infectious PEDV strains. To construct the full-length cDNA clone of the PEDV strain MSCH, an intermediate BAC plasmid, pBAC-M-PEDV, was generated. Five overlapping DNA fragments (A-E) were subsequently amplified and assembled into pBAC-M-PEDV (Figure 1A). The pBAC-PEDV MSCH plasmids were subsequently transfected into Vero cells, and the CPEs, characterized by syncytium formation, were observed after 36 h. The rescued wild-type recombinant PEDV (rPEDV-WT) was further identified by indirect immunofluorescence assay (Figure 1B) and western blot (Figure 1C) using a mouse anti-PEDV N monoclonal antibody. Sanger sequencing data and restriction site analysis revealed that the silent T1810C substitution introduced in the PEDV infectious cDNA that generates a Sac II restriction site was recovered in rPEDV-WT (Figure 1D). This mutation is a silent mutation and does not affect the encoded protein at this position. The nucleic acid electrophoresis results also indicated that the rescued virus had an additional Sac II restriction site at this position (Figure 1D). Viral growth kinetics and plaque morphology assays demonstrated that rPEDV exhibited similar growth characteristics to the parental strain, confirming successful rescue (Figures 1E and F).

### Rescue of PEDVs with mutations in the N-glycosylation site in the S protein

**Figure 2:**
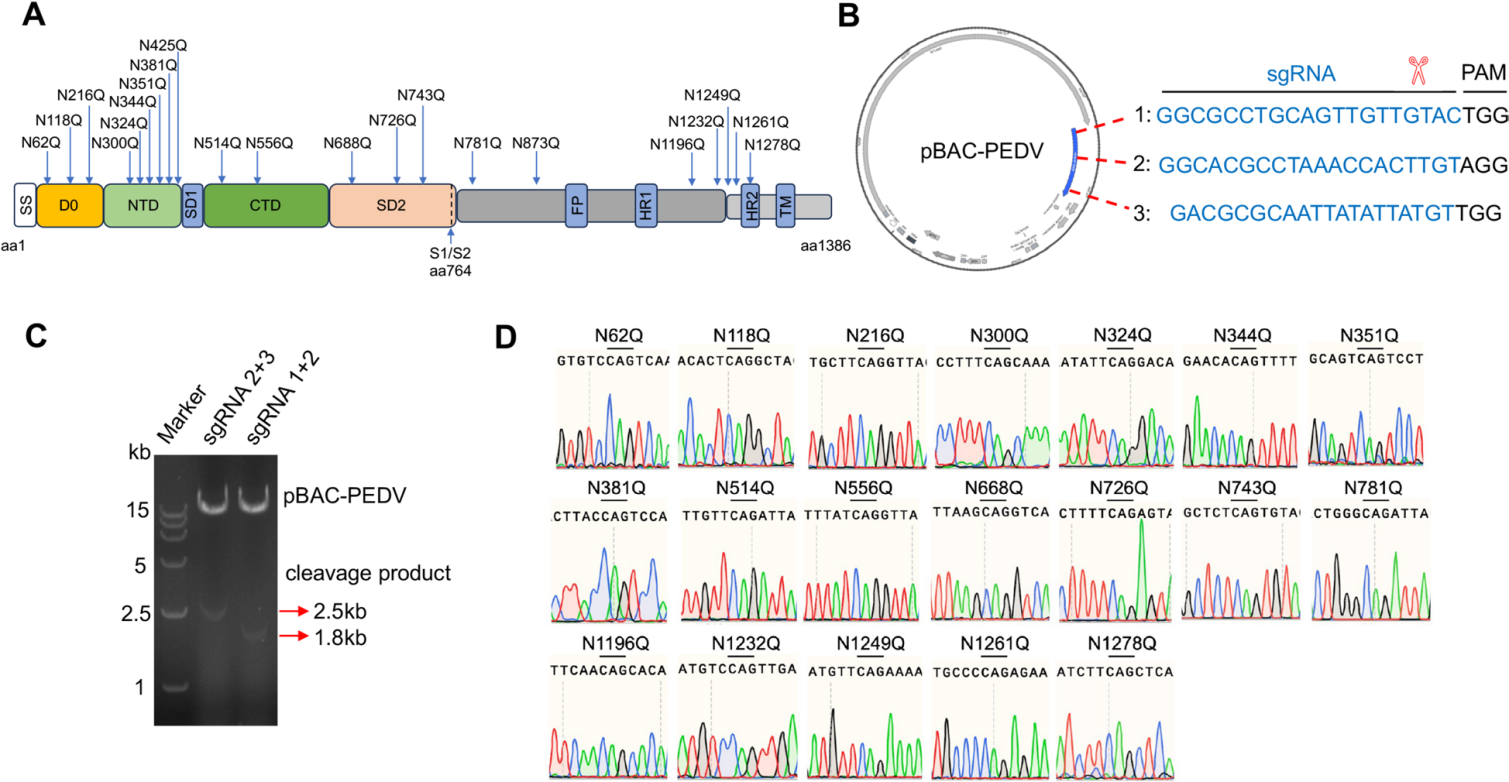
**Rescue of PEDVs with S protein N-glycosylation site mutations.** **A** Illustration of the mutants at the putative glycosylation sites (21 sites). **B** Schematic diagram of the cleavage of pBAC-PEDV with CRISPR/Cas9. The nt positions correspond to the complete genome sequence of MSCH. **C** Electrophoresis of the cleaved pBAC-PEDV in a 0.8% agarose gel to verify specific cleavage. The expected cleavage products were approximately 2.5 kb and 1.8 kb in length. **D** The 19 successfully rescued mutant viruses were passaged to the 5^th^ generation for sequencing to assess their passage stability.

Considering the crucial role of glycosylation in the S protein of coronaviruses in viral replication, we next sought to investigate the functional significance of N-glycosylation sites in the S protein. To this end, we first predicted the 21 N-glycosylation sites of the S protein in the PEDV strain MSCH using NetNGlyc-1.0 (Figure 2A). To facilitate the generation of mutant viruses, we modified the PEDV genomic cDNA in the BAC plasmid using CRISPR/Cas9 technology. Three single guide RNA (sgRNA1, sgRNA2 and sgRNA3) were designed to target and cleave the S gene (Figure 2B). As shown in Figure 2C, pBAC-PEDV was cleaved at the designed sites, producing a linearized BAC and cleavage product fragment.

The glycosylation mutant plasmids were then transfected into Vero cells, the CPE analysis revealed that of the 21 infectious clone plasmids, 19 PEDV mutants were successfully rescued, whereas mutations at N425Q and N873Q were lethal to the virus (Table 2). These findings indicate that these two glycosylation sites are indispensable for viral viability. Sequencing data confirmed the successful replacement of asparagine (N) with glutamine (Q) at the targeted sites (Figure 2D). These results indicate that we successfully constructed 19 PEDV mutants, each carrying a single N-glycosylation KO mutation in the S protein.

**Table 2:** **PEDV BAC recovery rates in cells**

BAC	No. of viruses recovered/no. of attempts	CPE	Western blot	Recovery (%)
rPEDV-S-N62Q	3/3	Yes	Yes	100
rPEDV-S-N118Q	3/3	Yes	Yes	100
rPEDV-S-N216Q	3/3	Yes	Yes	100
rPEDV-S-N300Q	3/3	Yes	Yes	100
rPEDV-S-N324Q	3/3	Yes	Yes	100
rPEDV-S-N344Q	3/3	Yes	Yes	100
rPEDV-S-N351Q	3/3	Yes	Yes	100
rPEDV-S-N381Q	3/3	Yes	Yes	100
rPEDV-S-N425Q	0/3	No	No	0
rPEDV-S-N514Q	3/3	Yes	Yes	100
rPEDV-S-N556Q	3/3	Yes	Yes	100
rPEDV-S-N688Q	3/3	Yes	Yes	100
rPEDV-S-N726Q	3/3	Yes	Yes	100
rPEDV-S-N743Q	3/3	Yes	Yes	100
rPEDV-S-N781Q	3/3	Yes	Yes	100
rPEDV-S-N873Q	0/3	No	No	0
rPEDV-S-N1196Q	3/3	Yes	Yes	100
rPEDV-S-N1232Q	3/3	Yes	Yes	100
rPEDV-S-N1249Q	3/3	Yes	Yes	100
rPEDV-S-N1261Q	3/3	Yes	Yes	100
rPEDV-S-N1278Q	3/3	Yes	Yes	100

### Impact of N-glycosylation of the S protein on viral replication

**Figure 3:**
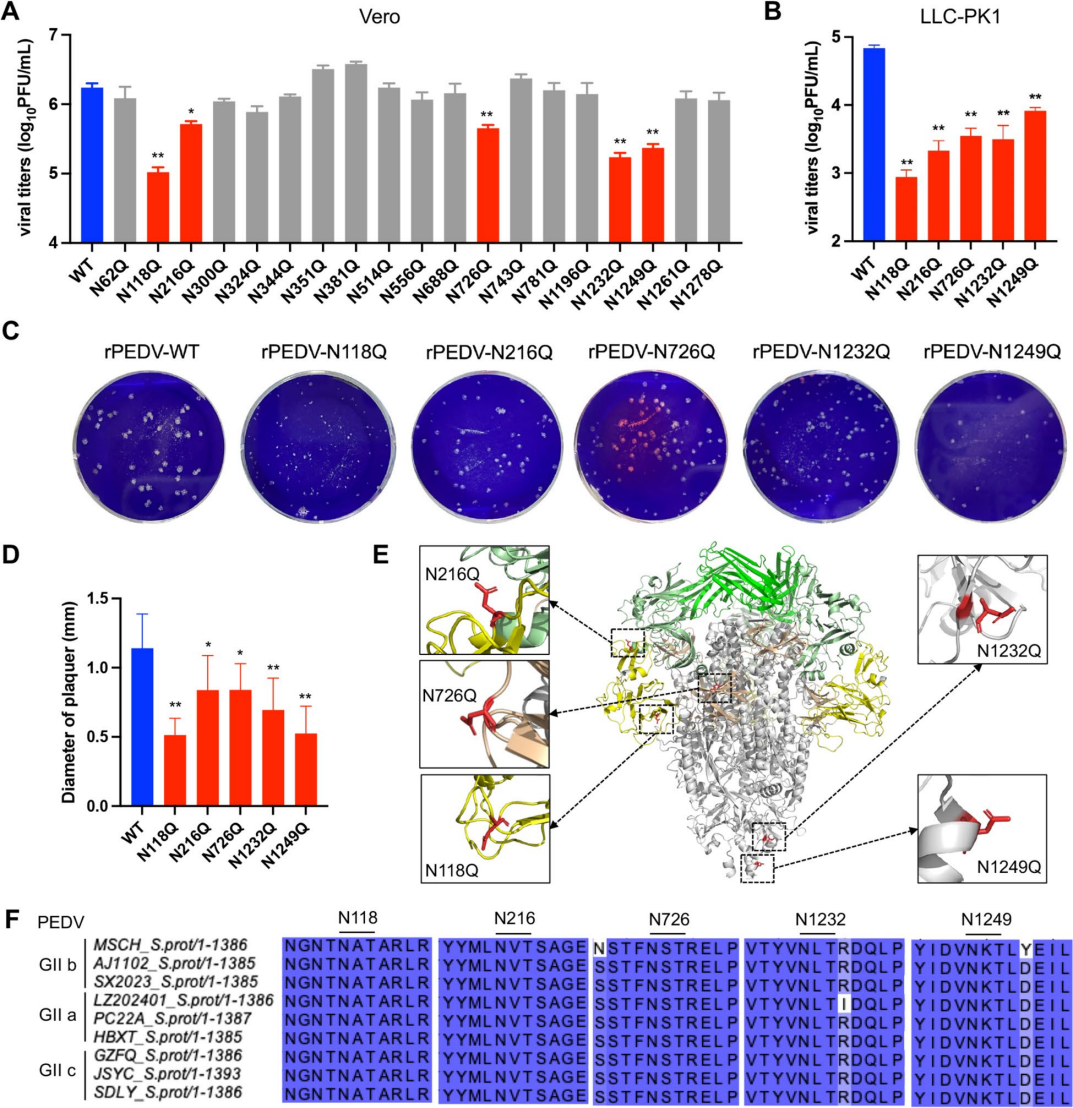
**Infectivity analysis of deletions of the glycosylation site mutants in vitro.** **A** Vero cells cultured in 6-well plates were infected with rPEDV-WT (WT) or 19 different S mutants (MOI of 0.01). The samples were collected at 24 hpi for titre determination. **B** Infectivity of 5 mutants (N118Q, N216Q, N726Q, N1232Q and N1249Q) in LLC-PK1 cells. **C** Representative images of the plaque morphologies of mock and PEDVs. **D** The size of the plaque was calculated using ImageJ software. **E** The relative positions of the 5 glycosylation sites on the 3D model of the S protein. **F** Amino acid sequences of the different GII subtypes at the five N-linked glycosylation sites.

To determine the infectivity of these mutants, we first infected Vero cells with 19 glycosylation deletion viruses and wild-type virus. Through a viral titre assay, mutations at N118, N216, N726, N1232, and N1249 significantly reduced viral titres compared with those of the wild-type virus (Figure 3A). The reduced infectivity of the five mutants was further confirmed in the LLC-PK1 cell line (Figure 3B). Plaque morphology assays demonstrated that these mutants formed smaller plaques than did the wild-type virus (Figures 3C and D). Additionally, to assess whether these N-glycosylation sites modulate S protein stability, we transiently transfected S protein mutants with mutations at these five glycosylation sites. Western blot analysis demonstrated that these mutations did not alter the steady-state protein levels compared with those of the WT S protein (Additional file 1). To visualize these glycosylation sites, the locations of these glycosylation sites were mapped onto a 3D model of the S protein trimer, and the five N-glycosylation sites (N118, N216, N726, N1232, and N1249) are highlighted with red sticks (Figure 3E). A comparison of the five glycosylation sites between the G2b strain MSCH and current circulating G2a/G2c strains revealed that they are conserved across these genotypes (Figure 3F). Overall, of the 19 glycosylation mutant viruses, the deletion of glycosylation at site 5 significantly reduced viral infectivity in vitro.

### Effect of N-glycosylation of the S protein on PEDV replication cycle

**Figure 4:**
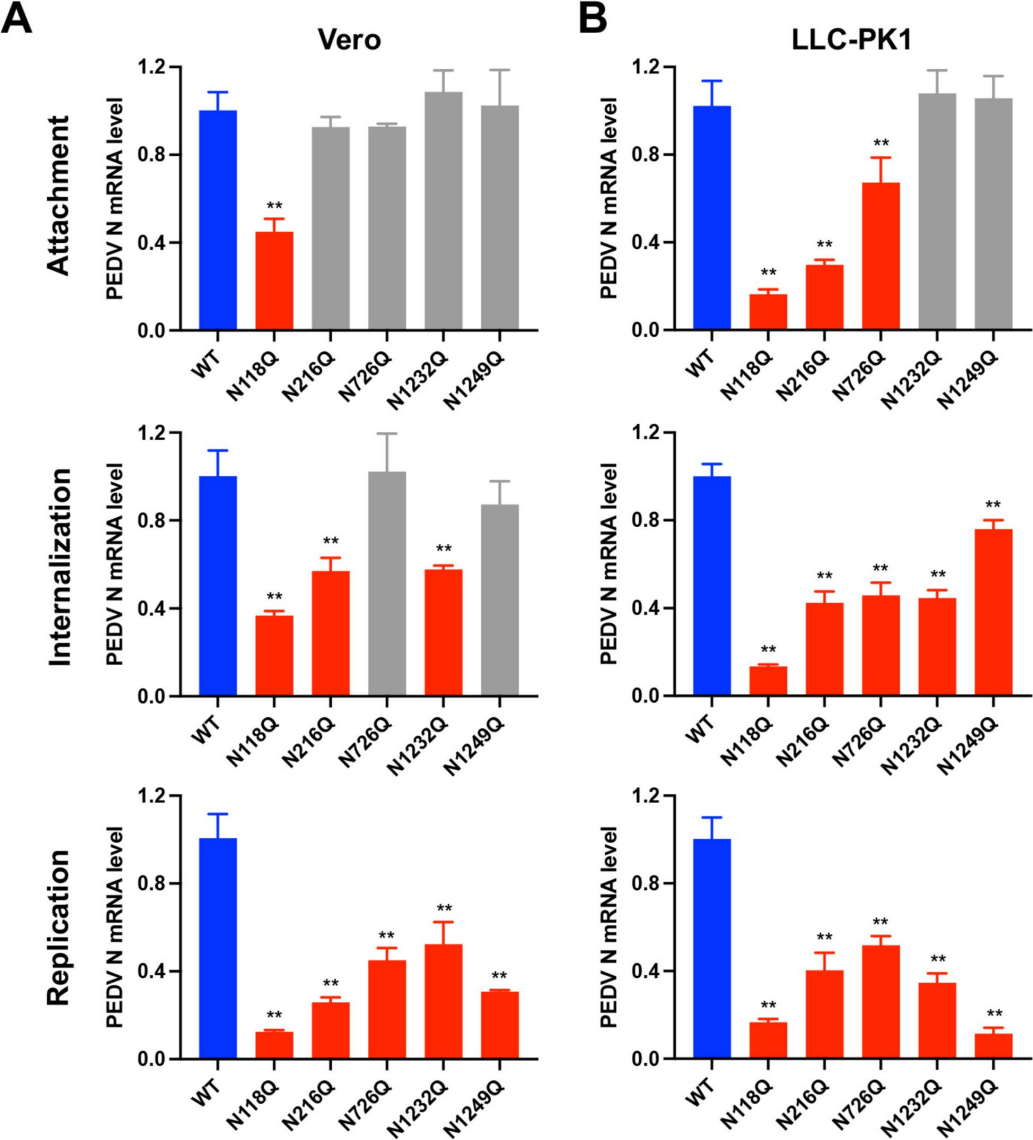
**Impact of glycosylation site mutations on the PEDV replication cycle.** Replication cycles of the glycosylation site mutant viruses and the parental virus were determined in Vero cells (**A**) and LLC-PK1 cells (**B**).

To determine the underlying mechanisms by which these mutations affect viral infectivity, we examined the impact of these mutations on viral attachment, internalization, and replication in Vero cells and pig LLC-PK1 cells. As shown in Figures 4A and 4B, in Vero cells, only the mutation at the N118Q site significantly affected viral attachment compared with the wild-type virus. However, in pig LLC-PK1 cells, mutations at the N118Q, N216Q, and N726Q sites affected viral attachment. By evaluating the impact of glycosylation site mutations on PEDV internalization, we found that in Vero cells, mutations at the N118Q, N216Q, and N1232Q sites affected viral internalization. In LLC-PK1 cells, all five mutations affected the process of viral entry into the cells. These findings suggest that N-glycosylation plays a critical role in the early stages of viral infection.

### Pathogenicity of PEDV mutants in piglets

**Figure 5:**
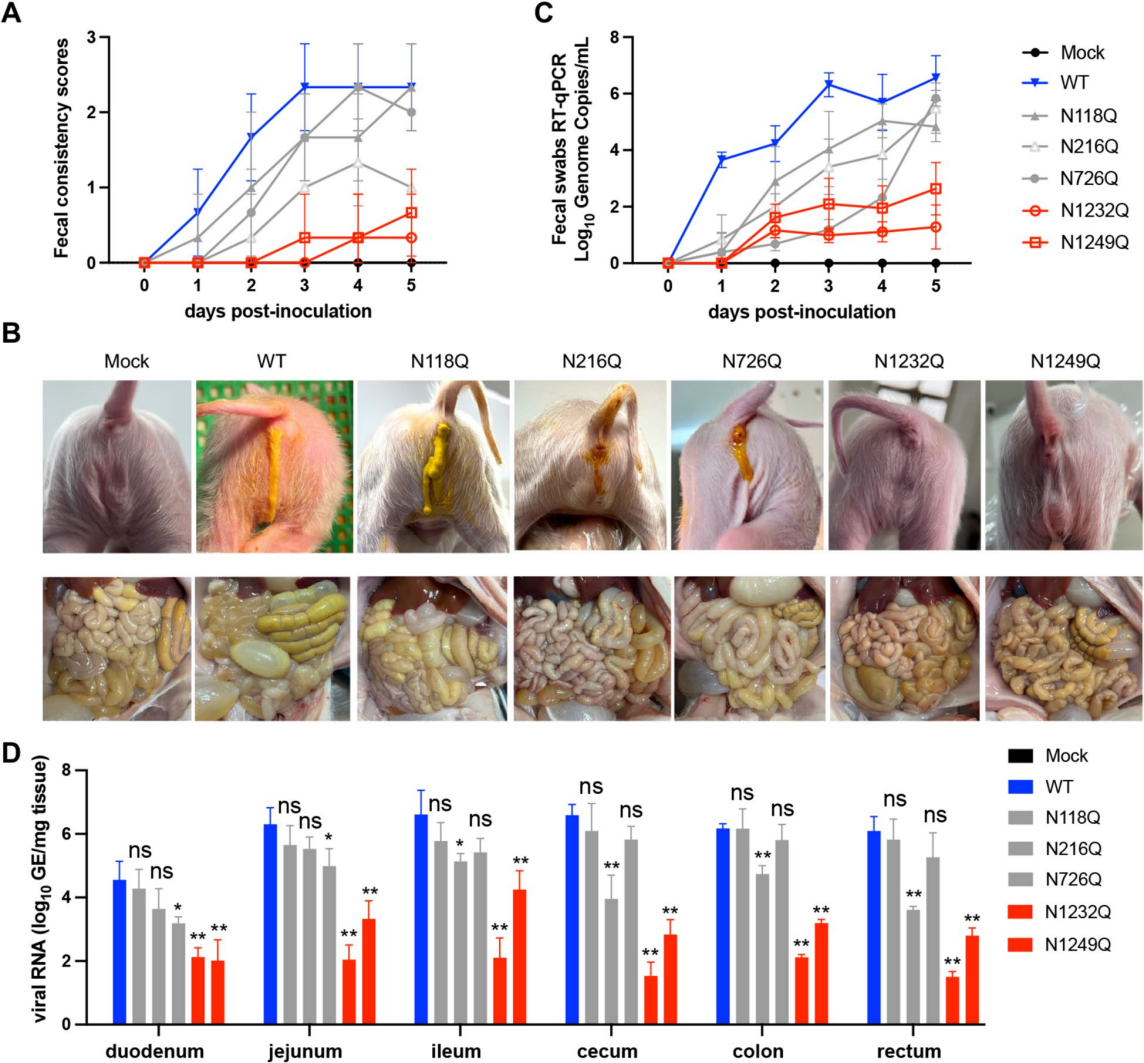
**Pathogenicity of PEDV mutants in piglets.** **A** Evaluation of the fecal consistency of piglets. Fecal consistency scoring: 0 (solid), 1 (pasty), 2 (semiliquid), and 3 (liquid). **B** Representative clinical signs and gross examination of piglets. **C** Detection of viral RNA in faecal swabs by RT-qPCR. **D** Viral RNA loads per milligram of intestinal tissue sample from infected piglets at 5 days post-challenge.

Next, we evaluated the pathogenicity of PEDV glycosylation mutants by oral inoculation with WT, N118Q, N216Q, N726Q, N1232Q and N1249Q. A portion of piglets infected with WT virus started to display diarrhea at 20 h post-challenge (hpc), and all the piglets presented diarrhoea accompanied by vomiting 2 days post-challenge (dpc) (Figures 5A and B). At necropsy, the intestinal walls of the WT-infected piglets were thin and flaccid, accompanied by a large amount of diffuse, mucoid, and yellowish digesta (Figure 5B). In the N118Q, N216Q, and N726Q challenged groups, two piglets presented pasty faeces during the 36–48 hpc period, but all piglets developed mild diarrhea by 3 dpc. Notably, piglets infected with N1232Q or N1249Q presented normal feces and occasionally pasty feces. Necropsy revealed no morphological changes in the intestines of the piglets (Figure 5B).

Rectal swabs collected from the challenged piglets were subjected to viral RNA extraction and RT-qPCR. As shown in Figure 5C, piglets infected with the WT virus presented the highest level of fecal RNA at 3 dpc, whereas the N1232Q and N1249Q groups presented extremely low viral RNA levels in the fecal swabs. To further validate viral pathogenicity, we measured the viral loads across distinct intestinal tissues (duodenum, jejunum, ileum, cecum, colon and rectum) of the infected piglets. As shown in Figure 5D, higher viral RNA loads were observed in the intestinal tissues of the WT, N118Q, N216Q and N726Q groups; in contrast, the viral RNA loads in the N1232Q and N1249Q groups were consistently lower across all tissue samples.

**Figure 6:**
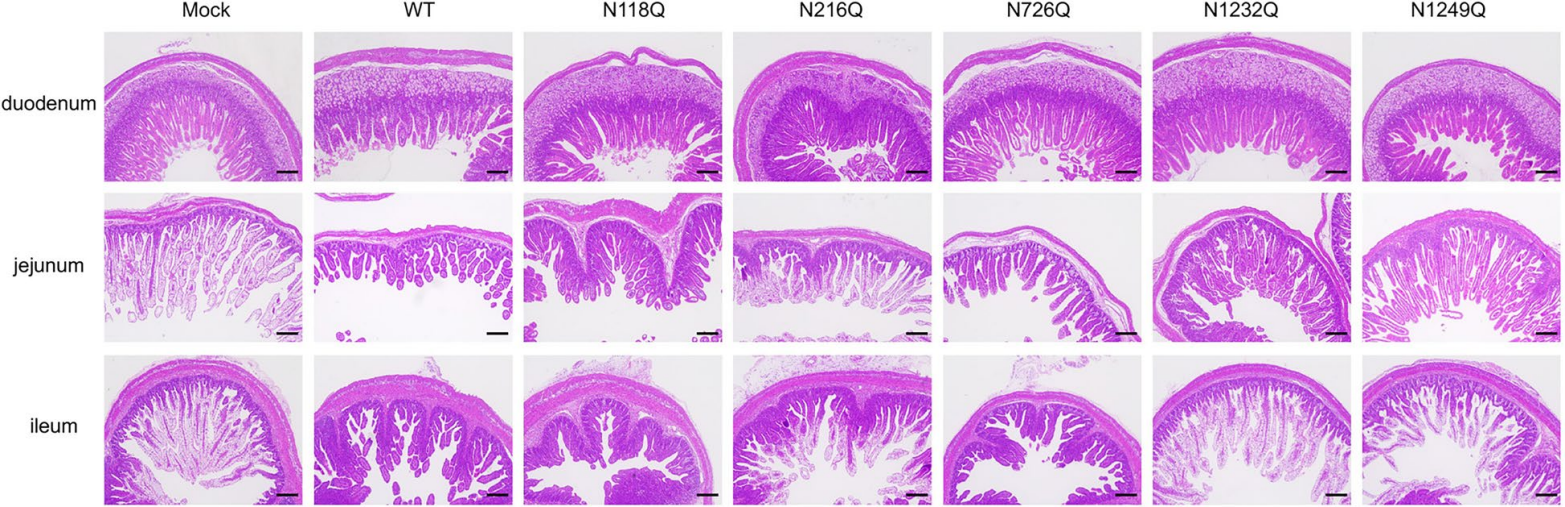
**Histopathological examination of the intestines of PEDV mutant-infected piglets.** Piglets were challenged with PEDVs and euthanized at 5 dpc. Various segments of the intestine, including the duodenum, jejunum, ileum, cecum, colon, and rectum, were collected and prepared for H&E staining. Scale bar, 200 µm.

**Figure 7:**
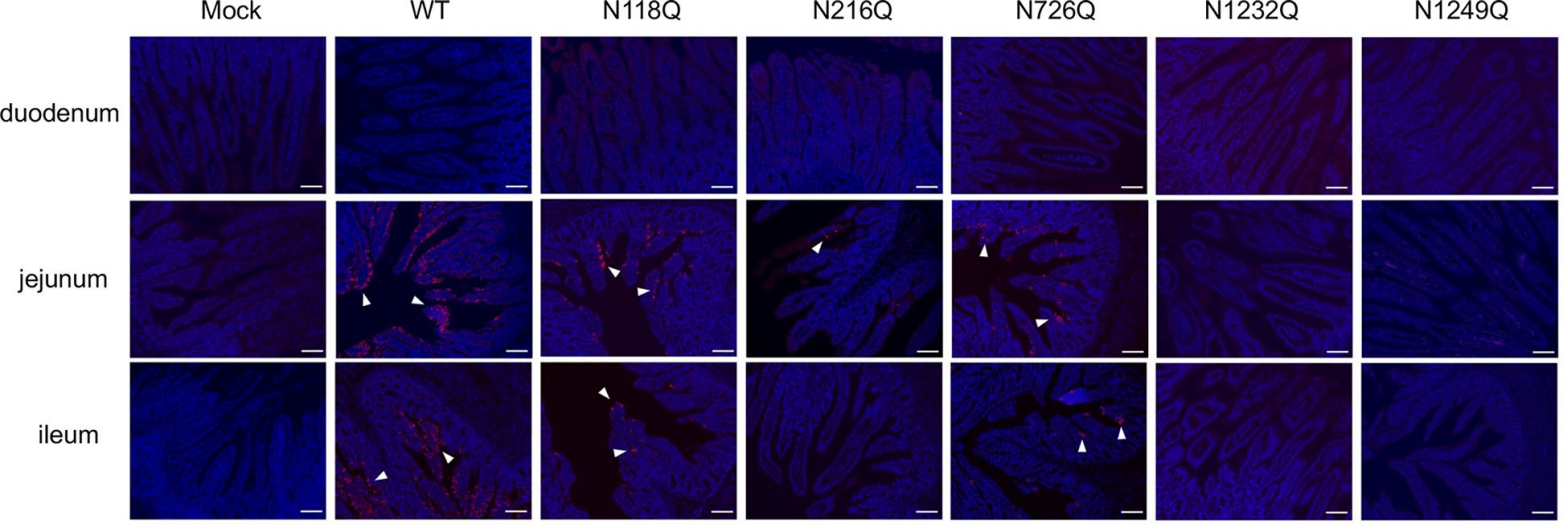
**Detection of the PEDV N antigen in intestinal sections.** Immunofluorescence findings in intestine sections of piglets euthanized at 5 dpc. PEDV N was labelled with red fluorescence, and the cell nuclei were stained blue with DAPI. The arrow indicates the location of the PEDV antigen. Scale bar, 100 µm.

Furthermore, histopathological examination revealed severe villous atrophy in the intestines of wild-type-infected piglets, whereas the N1232Q and N1249Q groups presented no significant pathology (Figure 6). IFA assays confirmed the presence of PEDV antigens in the intestines of wild-type-infected piglets but not in those infected with the N1232Q and N1249Q mutants (Figure 7). Collectively, assessments of clinical symptoms, viral RNA loads and histopathology indicate that the recombinant viruses N1232Q and N1249Q exhibit low pathogenic phenotypes.

## Discussion

PEDV infection is one of the major causes of diarrhea in piglets, causing significant economic losses to the global pig farming industry [38]. Therefore, exploring protective strategies against PEDV is of paramount importance. The coronavirus S protein is a multifunctional protein that plays various roles in the virus life cycle. N-linked glycosylation is a common glycosylation found in enveloped viruses that contributes to protein structure and function. Previous studies have demonstrated that N-glycosylation plays a critical role in the infectivity and pathogenicity of various coronaviruses. For example, Zan et al. [39] reported that N-glycosylation of the S2 subunit stabilizes the prefusion conformation of the SARS-CoV-2 spike protein, thereby facilitating viral entry. Here, we used a reverse genetics system to generate N-glycosylation-defective recombinant viruses and demonstrated that N-glycosylation of the PEDV S protein affects viral replication and pathogenicity.

We obtained 19 N-glycosylation mutant viruses by mutating the glycosylation sites in the S protein and rescuing the viruses. The failure to rescue the N425Q and N873Q mutants highlights their critical role in maintaining the fundamental structure or function of the S protein, which is possibly involved in essential processes such as viral entry or genome replication. When the replication differences of glycosylation-deficient viruses at the cellular level were tested, five of the N-glycosylation mutant viruses exhibited significantly reduced replication. Western blot analysis revealed that the five glycosylation site mutations did not significantly affect S protein stability. Notably, treatment with swainsonine (a Golgi glycosylation inhibitor) abolished the upper band corresponding to the Golgi-processed mature S protein, leaving only the ER-resident precursor band (Additional file 2), confirming that the two distinct bands observed in our study represented different glycoform maturation stages.

Since the S protein plays a crucial role in the viral entry process, we examined the effects of glycosylation site mutations on the viral replication cycle in Vero cells and pig LLC-PK1 cells. As expected, these five glycosylation site mutations were associated with a relatively slow rate of viral entry, especially the mutation at the N118 site. This could be attributed to conformational changes in the S protein trimer, thereby affecting the virus's binding to cell receptors [30, 40, 41]. Interestingly, we found that the differences in pig LLC-PK1 cells were more pronounced than those in Vero cells, which may be due to differences in the viral entry mechanisms across different cell types. According to the report by Zan et al., among the 22 N-linked glycosylation sites, 9 are crucial for the folding and maturation of the SARS-CoV-2 S protein [39]. Further experiments are needed to confirm whether the five glycosylation mutations affect the maturation of the PEDV S protein.

Previous studies by Ma et al. identified five N-glycosylation sites in the D0 and SD2 domains of the PEDV spike protein as critical for virulence evolution, demonstrating that mutations at positions 62 and 722 attenuated virulence in piglets while preserving immunogenicity [42]. These findings align with our findings that N-linked glycosylation influences spike protein function and viral pathogenesis. While Ma et al. focused on glycosylation sites differentiating GI and GII subtypes, our systematic mutational analysis of 21 predicted N-glycosylation sites revealed additional critical sites. This study selected five glycosylation mutant viruses with reduced replication levels in vitro to assess the impact of glycosylation mutations on the pathogenicity of PEDV. Piglets infected with N1232Q or N1249Q presented milder symptoms than those infected with wild-type PEDV did, making potential candidates for live attenuated PEDV vaccine design. However, the immunogenicity of the virus in vivo remains to be determined. Additional studies have shown that the S protein of coronaviruses can impede the transcription of interferon-stimulated genes (ISGs) by disrupting the formation of the interferon-stimulated gene factor 3 (ISGF3) complex, thus dampening the host's antiviral immune response [43, 44]. We believe that it would be important to explore whether the mutations N1232Q and N1249Q impact the S protein’s capacity to inhibit interferon in follow-up studies. Additionally, we found that the replication capacity of the mutant viruses in vitro was not always positively correlated with the clinical symptoms they caused. This may be because the pathogenicity of the mutant viruses is also related to their ability to colonize the intestinal compartments.

In summary, our data indicate that among the 21 predicted N-linked glycosylation sites in the PEDV S protein, mutations at 5 sites resulted in attenuated replication at the cellular level via perturbation of the viral entry process. Furthermore, the N-glycosylation-deficient viruses N1232Q and N1249Q exhibited reduced colonization in the piglet intestine and significantly lower pathogenicity than did the wild-type virus. This study deepens our understanding of PEDV pathogenicity and provides a new strategy for developing attenuated live vaccine candidates against PEDV.

## Supplementary Information

**Additional file 1. The impact of N-glycosylation site mutations on the stability of the S protein.** 293T cells were transfected with plasmids expressing Flag-tagged WT or mutant S proteins (N118Q, N216Q, N726Q, N1232Q, and N1249Q). At 24 h post-transfection, the cells were treated with CHX (100 μg/mL), and the lysates were collected at 3 and 6 h post-treatment. Western blot analysis was performed using an anti-Flag antibody. β-actin was used as a loading control.

**Additional file 2. Validation of S protein maturation status via Golgi glycosylation inhibitors.** 293T cells transfected with Flag-tagged S protein were treated with swainsonine (2 μM and 20 μM) or DMSO. Lysates were analysed by western blotting using an anti-Flag antibody. β-actin was used as a loading control.

## Data Availability

All the data generated or analysed during this study are included in this published article.
