# Supplementary figures and images for: N-glycosylation of the PEDV spike protein modulates viral replication and pathogenicity
Source: Vet Res. 2025 Aug 29;56:172. doi: 10.1186/s13567-025-01606-9 (PMC12395675; doi:10.1186/s13567-025-01606-9)

**
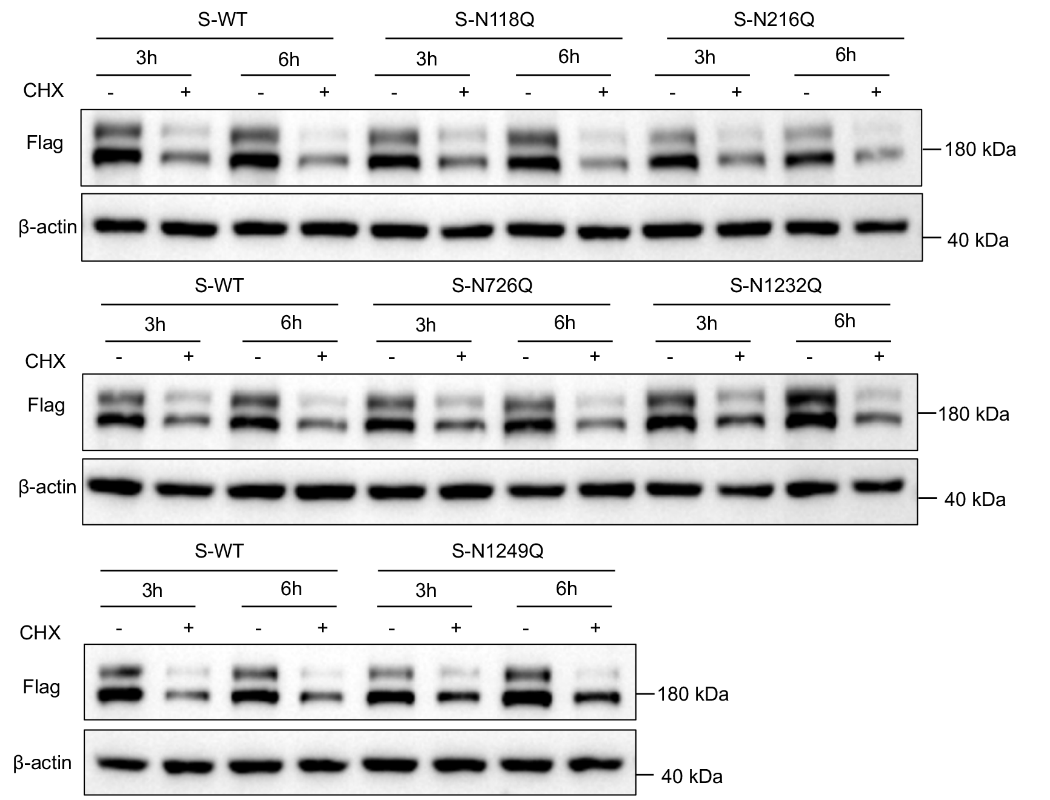
**

Supplement: Supplementary file 1 — Additional file 1. The impact of N-glycosylation site mutations on the stability of the S protein. 293T cells were transfected with plasmids expressing Flag-tagged WT or mutant S proteins (N118Q, N216Q, N726Q, N1232Q, and N1249Q). At 24 h post-transfection, the cells were treated with CHX (100 μg/mL), and the lysates were collected at 3 and 6 h post-treatment. Western blot analysis was performed using an anti-Flag antibody. β-actin was used as a loading control. [file 13567_2025_1606_MOESM1_ESM.docx]

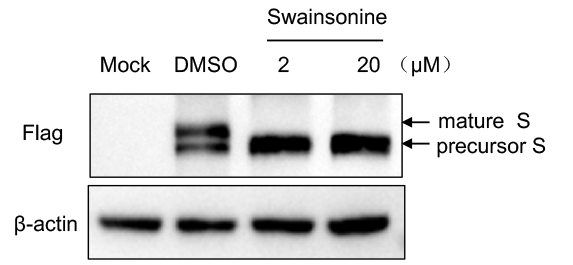

Supplement: Supplementary file 2 — Additional file 2. Validation of S protein maturation status via Golgi glycosylation inhibitors. 293T cells transfected with Flag-tagged S protein were treated with swainsonine (2 μM and 20 μM) or DMSO. Lysates were analysed by western blotting using an anti-Flag antibody. β-actin was used as a loading control. [file 13567_2025_1606_MOESM2_ESM.docx]
